# Krill Oil Has Different Effects on the Plasma Lipidome Compared with Fish Oil Following 30 Days of Supplementation in Healthy Women: A Randomized Controlled and Crossover Study

**DOI:** 10.3390/nu12092804

**Published:** 2020-09-13

**Authors:** Hyunsin H. Sung, Andrew J. Sinclair, Kevin Huynh, Adam A. T. Smith, Natalie A. Mellett, Peter J. Meikle, Xiao Q. Su

**Affiliations:** 1 Institute for Health and Sport, Victoria University, P.O. Box 14428, Melbourne, VIC 8001, Australia; hyunsin.sung@live.vu.edu.au; 2 Faculty of Health, Deakin University, Geelong, VIC 3217, Australia; 3 Department of Nutrition, Dietetics and Food, Monash University, Notting Hill, VIC 3168, Australia; 4 Metabolomics Laboratory, Baker Heart and Diabetes Institute, Melbourne, VIC 3004, Australia; kevin.huynh@baker.edu.au (K.H.); alexander.smith@baker.edu.au (A.A.T.S.); natalie.mellett@baker.edu.au (N.A.M.)

**Keywords:** krill oil, fish oil, plasma lipidomic response, EPA, DHA, phospholipids, phosphatidylcholine, 30-day study, women, lipidome

## Abstract

This is a follow-up of our previous postprandial study and it focused on the plasma lipidomic responses to 30 days of krill oil (KO) versus fish oil (FO) supplementations in healthy women. Eleven women (aged 18–50 years) consumed KO or FO for 30 days in a randomized, cross-over study, with at least a four-week washout period between supplementations. The daily supplements provided 1.27 g/day of long-chain (LC) omega-3 polyunsaturated fatty acids (PUFA) from KO (containing 0.76 g eicosapentaenoic acid (EPA), 0.42 g docosahexaenoic acid (DHA)) and 1.44 g/day from FO (containing 0.79 g EPA, 0.47 g DHA). Fasting plasma samples at days 0, 15, and 30 were analyzed using gas chromatography and liquid chromatography electrospray ionisation-tandem mass spectrometry. KO resulted in a significantly greater relative area under the curve (relAUC) for plasma EPA after 30 days. Lipidomic analysis showed that 26 of 43 lipid molecular species had a significantly greater relAUC in the KO group, while 17/43 showed a significantly lower relAUC compared with the FO group. More than 38% of the lipids species which increased more following KO contained omega-3 PUFA, while where FO was greater than KO, only 12% contained omega-3 PUFA. These data show that KO and FO do not have equivalent effects on the plasma lipidome.

## 1. Introduction

Krill oil (KO) is a novel source of long-chain omega-3 polyunsaturated fatty acids (LC omega-3 PUFA), in which eicosapentaenoic acid (EPA) and docosahexaenoic acid (DHA) are mainly in phosphatidylcholine (PC), while in fish oil (FO) the LC omega-3 PUFA are in triacylglycerol (TG) [1,2,3,4]. Some studies report increased plasma levels of omega-3 PUFA from KO versus FO, although other studies have reported no significant differences between the two supplements [5,6,7].

The inconsistent outcomes from the above studies might be related to the limitations of study designs, insufficient control of the food matrix and lipid content of the test meal consumed by study participants, different dose rates, and different chemical forms of LC omega-3 PUFA [8,9].

Other biochemical features of KO that might contribute to differences in health outcomes compared with FO include the presence of astaxanthin and choline (as phosphatidylcholine, (PC)). Indeed, a recent study showed that the choline from KO was equally bioavailable to free choline but with a later peak absorption [10].

There are several studies that have demonstrated changes of plasma lipidomes following LC omega-3 PUFA intakes [11,12]. However, only one study has compared the plasma lipidomes in response to KO and FO in humans; this was a postprandial study which showed that EPA and DHA from KO were preferentially incorporated into phospholipid molecular species, whereas after FO consumption, these fatty acids were preferentially partitioned to neutral lipid species [1]. The present study is a follow-up of our previous postprandial investigation and is the first longer-term study examining lipidomic profiles in subjects supplemented with KO and FO. To minimize variability in study participants, healthy young women were recruited for this randomized, cross-over study with at least a four-week washout period between the two marine oil supplementations. A single gender was chosen to eliminate gender variation, particularly for sex hormone effects on lipid metabolism. In addition, evidence of drug and/or supplement benefit to women is limited because clinical trials have focused more on men than women, who are often underrepresented in clinical trials [13]. This was because women often experience more side effects from new treatments. Also, menopause cycle and hormone level changes could cause variability. Women respond differently from men to a broad array of treatments; therefore, it was important to investigate the health impact of supplements like KO on the female population. It was hypothesized that 1.27 g/day of LC omega-3 PUFA from KO supplementation, compared with 1.44 g/day of LC omega-3 PUFA from FO (the closest possible match to these fatty acids from the commercial capsules), would result in differences in plasma lipidomic response between the two supplements. Since FO has been commonly used as a supplement in a large number of studies, the objective of this study was to compare KO with FO supplementation.

## 2. Materials and Methods

### 2.1. Study Participants

A total of 11 healthy premenopausal women aged between 18 and 50 years within BMI 20–35 (kg/m^2^) completed the study. They were recruited from Victoria University staff and students, community centers, and local medical practices. Participants were screened for their suitability for the study using a medical questionnaire, anthropometric measurements, and were excluded if their daily LC omega-3 PUFA was more than 500 mg based on results of the electronic PUFA Food Frequency Questionnaire (FFQ) [14]. Participants were also excluded if they were cigarette smokers; pregnant or lactating; had heart, liver, kidney, or inflammatory bowel disease; diabetes; medications interfering with lipid metabolism or lowering blood lipids; allergy to fish or seafood; or had consumed oily fish more than twice a week or supplements including omega-3 fatty acids in the past four weeks prior to the study. The study was undertaken at Victoria University Metabolic Clinical Laboratory, St Albans campus.

### 2.2. Study Design

**Figure 1:**
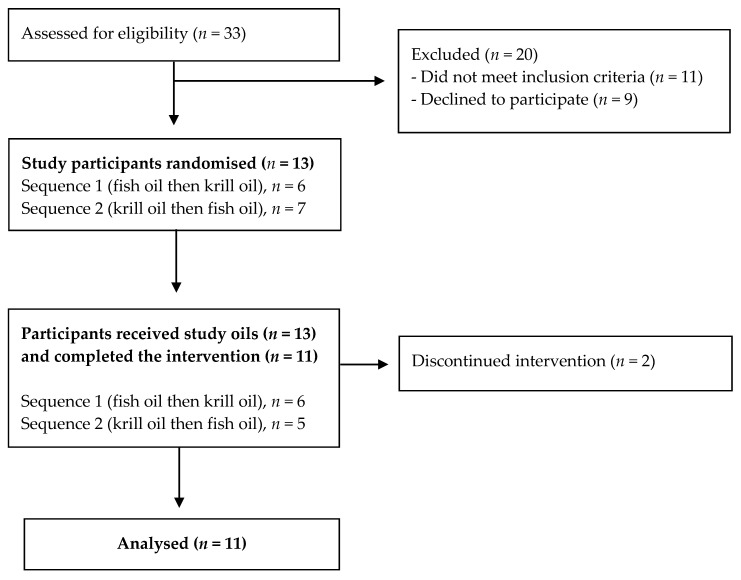
Study design chart *n* = the number of participants. Each subject received each of the two supplementations (krill oil or fish oil) in randomized order, as shown in the flow chart. There was at least a 4-week washout between two supplementations.

This study was a randomized crossover study with KO and FO supplementation for 30 days and separated by a minimum washout period of 4 weeks between supplementations following studies by Hodge et al. [15] and Cicero et al. [16] (Figure 1). The two supplementations were allocated randomly by the principal investigator who was not directly involved in data collection. Participants were instructed to maintain their habitual diet and requested not to consume any food or supplements containing omega-3 PUFA more than once a week during the study periods. For interventions, participants consumed daily either 7 one-gram capsules of KO (*Euphausia superba* oil, Swisse Wellness Pty Ltd., Victoria, Australia) containing 1.27 g of LC omega-3 PUFA (0.76 g EPA, 0.42 g DHA, 0.09 g DPA) or 5 one-gram capsules of FO (Natural FO, Swisse Wellness Pty Ltd., Victoria, Australia) containing 1.44 g of LC omega-3 PUFA (0.79 g EPA, 0.47 g DHA, 0.18 g DPA) for 30 days each. Participants were required to attend the clinic three times for blood and data collection, at days 0 (baseline), 15, and 30 for each supplementation period for blood sample collection. Prior to each clinic visit, participants consumed a low-fat evening meal, and were advised to avoid drinking alcohol and strenuous physical activities, and to fast from 10 pm. On each study day, standardized procedures were performed where participants arrived at the clinic between 7 am to 9 am, and a fasting blood sample (10 mL) was collected via a venepuncture by a qualified phlebotomist. After the blood sample collection, all participants completed a 24-h dietary recall and an electronic PUFA FFQ. The study protocol was approved by the Ethics Committee of Victoria University Human Research (HRE15-031). Informed consent was obtained from all participants prior to the study. This trial was registered with the Australian and NZ Clinical Trial Registry (ACTRN 12615000472572).

### 2.3. Dietary Assessment

Dietary fat intake was assessed at baseline, days 15 and 30, particularly for LC omega-3 PUFA, using an electronic PUFA FFQ, which contains data from NUTTAB 2010. A 24-h dietary recall was also used to monitor individuals’ food intake, particularly for the days before each study visit day. Data were analyzed using FoodWorks version 8 (Xyris software, Kenmore Hills, QLD, Australia) combined with NUTTAB 2010 and AUSNUT 2013. The electronic PUFA FFQ provides automatic calculations of estimated daily PUFA intake based on individuals’ average food intake for the previous three months [14].

### 2.4. Plasma and Oil Fatty Acids Analysis

The KO and FO capsules used in this study were purchased from a local pharmacy and the analysis of fatty acid profile was performed using the gas chromatography (GC) prior to the commencement of intervention. For each study oil, five randomly chosen oil capsules were mixed and analyzed six times using GC. The single capsule fill weight was 1054 mg for KO and 1063 mg for FO, and these values were used to calculate the EPA, DPA, and DHA contents (Supplementary Table S1). The plasma and oil samples were analyzed for fatty acid content as described previously [1], followed by gas liquid chromatography with a BPX70 capillary column. Both plasma samples and oil samples included an internal standard, tricosanoic acid, C23:0 (Nu-Chek Prep, Inc., Elysian, MN, USA). The KO and FO were typical of such oils, as reported in the literature, and contained 18% and 29% of total LC omega-3 PUFA, respectively. The omega-3 PUFA contents of the two oils are outlined here, as described previously [1]. KO, 7 capsules/day contained in mg/day: 759 mg EPA, 94 mg DPA, 417 mg DHA; FO, 5 capsules/day, contained 786 mg EPA, 182 mg DPA, and 473 mg DHA (Supplementary Table S1).

### 2.5. Lipid Extraction, Lipidomics Analysis, and Identification of Lipid Molecular Species

These details have been described fully in Sung et al. [1]. However, briefly, plasma samples were extracted in CHCl3:MeOH (2:1) together with an internal standard mix containing non-physiological or stable isotope-labelled lipid standards, as previously described [17]. Lipidomic analysis was performed by UHPLC ESI-MS/MS, using an Agilent 1290 HPLC coupled to an Agilent 6490 triple quadrupole mass spectrometer. Results from the chromatographic data were analyzed using Mass Hunter Quant where relative lipid abundances were calculated by relating the area under the chromatogram for each lipid species to the corresponding internal standard. Correction factors were applied to adjust for different response factors, where these were known [18]. Species that were chromatographically separated were labelled as such (e.g., PC(16:0–22:6) and PC(18:2–20:4)), whereas species that were mixed isomers were given the standard phospholipid notation (e.g., PC(40:8) was a mixture of 20:4/20:4 and 18:2–22:6) [18]. Where structural details were sufficient, lipids were manually annotated as containing long-chain omega-3 components (i.e., 20:5 EPA, 22:5 DPA, and 22:6 DHA).

### 2.6. Statistical Analysis

For each lipid species and lipid class, we performed two types of analyses to statistically evaluate changes over time. We calculated the ratio (expressed as fold change) between the area under the curve (AUC) delimited by lipid concentration across all available time points, and the AUC generated by assuming that the baseline lipid concentration remained constant (i.e., continued fasting). This “relative AUC” (relAUC) provides a single per-subject/oil estimate of the total 30-day lipid flux in plasma following supplementation. We then analyzed relAUCs between supplementations using paired *t*-tests for each species and class. In addition, we performed linear mixed modelling for each lipid class and species, wherein (log10) lipid concentration was modelled as being modulated by a combination of time, supplementation, and the interaction between the two, using subject as a random effect to account for subject-specific baseline variation [1].

*P*-value distributions were visually assessed for proper test behavior, and were corrected for multiple testing using Storey’s local false discovery rate procedure [19] using a multi-step strategy, detailed in our previous paper [1]. However, multiple testing correction remained very stringent, so we also report on nominal *p*-values for the lipid classes in this article. We considered corrected species-level and nominal class-level *p* < 0.05 as significant.

Lipid species and classes with at least one significant result in either analysis (relAUC or mixed models) were examined further. All analysis results, including descriptive summaries of lipid concentrations, are made available in Supplementary Tables S2 (lipid classes) and S3 (lipid species). All statistical analyses were carried out in R (x64, v. 3.5.0) (R Foundation for Statistical Computing, Vienna, Austria).

## 3. Results

### 3.1. Participant Characteristics and Intake of Dietary Long-Chain Omega-3 PUFA at Baseline

A total of 11 healthy premenopausal women completed the study. The average age of participants was 30.1 ± 7.1 years and body mass index was 25.0 ± 5.2 (kg/m^2^) at baseline. The LC omega-3 PUFA intake at baseline was 0.24 ± 0.15 g/day based on the PUFA FFQ. The mean interval between treatments was 41 days (range from 30 days to 75 days). Furthermore, we analyzed the baseline values for EPA and DHA at the start of each intervention on an individual basis to see if these values had returned to their original values; it was found there were no significant differences between the baseline values for either EPA or DHA (KO EPA baseline, 19.6 ± 3.2 µg/mL; FO EPA baseline, 16.7 ± 2.5 µg/mL, *p* = 0.399, and KO DHA baseline, 42.7 ± 4.1 µg/mL; FO DHA baseline, 38.6 ± 3.8 µg/mL, *p* = 0.229).

### 3.2. Responses of Plasma LC Omega-3 PUFA

**Figure 2:**
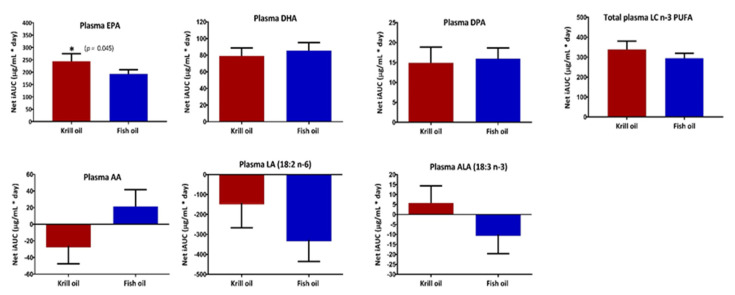
Relative AUCs of plasma LC omega-3 PUFA over the 30-day supplementations of KO and FO. Values are expressed as mean ± SEM. relAUC (µg/mL*day) from day zero to 30 (*n* = 11) of plasma fatty acids was calculated using the trapezoid rule and compared between the two supplementation groups using a paired *t*-test. *p* values are represented as * *p* < 0.05. Abbreviation: AA, arachidonic acid; ALA, alpha-linolenic acid; net relAUC, the incremental area under the curve from baseline to 30 days; EPA, eicosapentaenoic acid; DHA, docosahexaenoic acid; DPA, docosapentaenoic acid; LC n-3 PUFA, long-chain omega-3 polyunsaturated fatty acids; LA, linoleic acid.

After the 30-day dietary intervention, there was a significantly greater relAUC for plasma EPA after KO supplementation compared with FO supplementation (*p* = 0.045), with 23.9% higher levels being recorded at day 15 and 35.6% higher at day 30 (Figure 2). There were no significant differences in relAUC between the two supplementation groups for DHA, DPA, total LC omega-3 PUFA, arachidonic acid, linoleic acid, or alpha-linolenic acid.

### 3.3. Treatment-Dependent Changes in Lipid Classes

**Figure 3:**
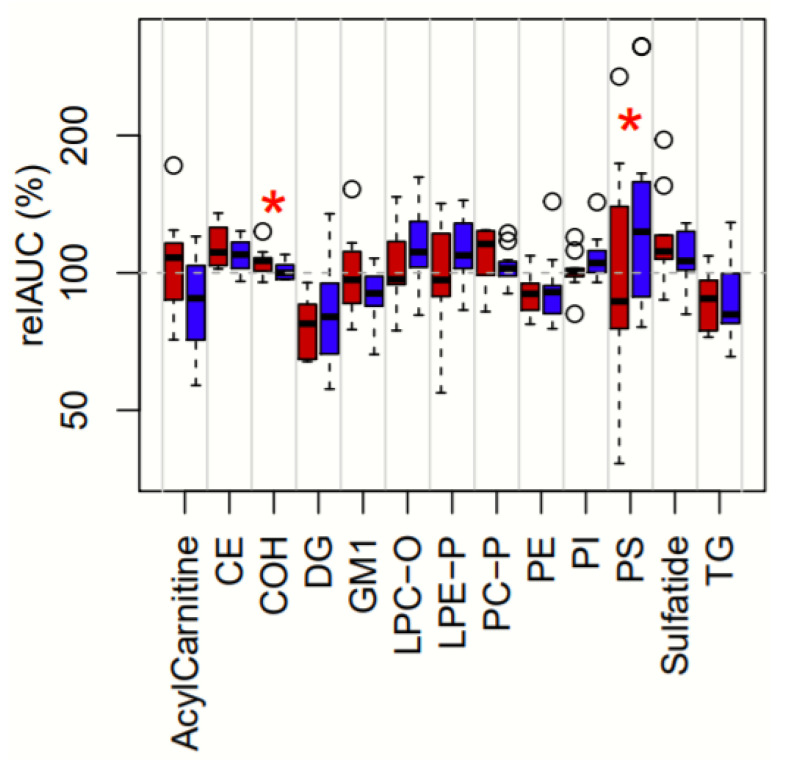
Relative AUCs following KO and FO supplementations for 13 lipid classes of interest. Thirteen lipid classes had at least one nominally significant change following at least one supplementation. Relative area under the curve (relAUC) is an estimate of the total plasma lipid flux over the 30-day period. Boxplots show the distributions of relAUC percentages across all 11 participants after KO (red) and FO (blue) supplementation. Asterisks (*) denote lipid classes with nominally significant (unadjusted *p* < 0.05) differences between treatments. Circles represent outlier values (outside of 1.5 times the interquartile range). Abbreviations: CE, cholesteryl ester; COH, cholesterol; DG, diacylglycerol; GM1, GM1 ganglioside; LPC-O, lysoalkylphosphatidylcholine; LPE-P, lysoalkenylphosphatidyethanolamine; PC-P, alkenylphosphatidylcholine; PE, phosphatidylethanolamine; PI, phosphatidylinositol; PS, phosphatidylserine; TG, triacylglycerol.

Thirteen of the 28 lipid classes had nominally significant supplementation-related changes after the 30-days (Supplementary Table S2). Figure 3 shows the distributions of their relAUCs following KO and FO. Two of these thirteen classes showed significantly different relAUCs between treatments; for cholesterol (COH) the KO was greater than FO, while for phosphatidylserine (PS) the FO was greater than KO. One lipid class, cholesterol ester (CE), had a higher concentration at the 15- or 30-day time point following KO supplementation compared with FO, while four lipid classes (DG, phosphatidylinositol (PI), alkyllysophosphatidylethanolamine, PS) had higher concentrations following FO supplementation compared with KO (Supplementary Table S2).

### 3.4. Changes in Lipid Species

A total of 520 molecular species, including those species containing LC omega-3 PUFA, were detected in 28 lipid classes (Supplementary Table S3), and 244 had values that were altered following supplementation (after multiple testing correction showing significantly different concentration, significant supplementation, and/or significant time × supplementation interaction).

**Figure 4:**
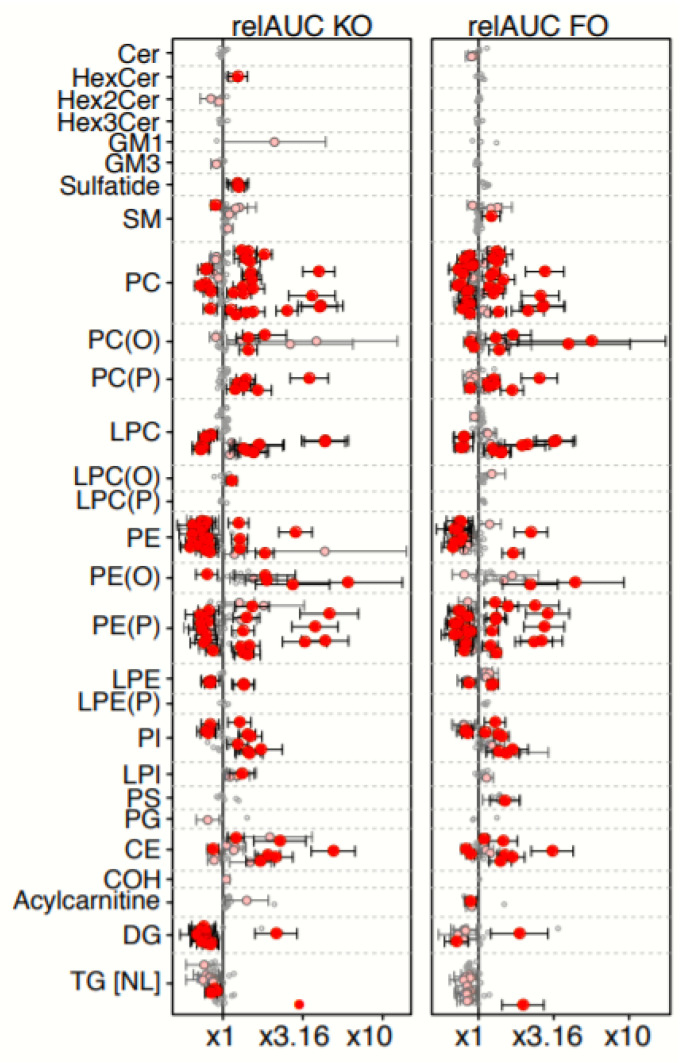
Relative AUCs of molecular lipid species following KO and FO supplementation for 30 days. Points show the mean relative area under the curve (relAUCs) for all molecular lipid species following KO (left) and FO (right) supplementation for 30 days. relAUC is an estimate of the total plasma lipid flux over the 30-day period. Values on the x-axis are expressed as (log-scaled) folds: a value of x1 indicates a constant lipid level over the course of the treatment, values above x1 indicate an overall gain, values below x1 indicate an overall loss. Dot colors: red = significant after multiple testing correction (adjusted *p* < 0.05, with 95% confidence intervals); pink = nominally significant (unadjusted *p* < 0.05, with 95% confidence intervals); grey = non-significant (no confidence intervals). Abbreviations: FO, fish oil; KO, krill oil; Cer, ceramide; HexCer, mono-hexosylceramide; Hex2Cer, di-hexosylceramide; Hex3Cer, tri-hexosylceramide; GM1, GM1 ganglioside; GM3, GM3 ganglioside; SM, Sphingomyelin; PC, Phosphatidylcholine; PC(O), alkylphosphatidylcholine; PC(P), alkenylphosphatidylcholine; LPC, lysophosphatidylcholine; LPC(O), lysoalkylphosphatidylcholine; LPC(P), lysoalkenylphosphatidylcholine; PE, phosphatidylethanolamine; PE(O), alkylphosphatidylethanolamine; PE(P), alkenylphosphatidylethanolamine; LPE, lysophosphatidylethanolamine; LPE-P, alkyllysophosphatidylethanolamine; PI, phosphatidylinositol; LPI, lysophosphatidylinositol; PS, phosphatidylserine; PG, phosphatidylglycerol; CE, cholesteryl ester; COH, cholesterol; DG: diacylglycerol; TG [NL], triacylglycerol (neutral loss feature).

As shown in Figure 4, for the 28 lipid classes examined, many species had relAUCs following the 30-day FO treatment showing significant (nominal or even multiple testing-corrected) deviations away from x1 (values above x1 indicate an overall gain, values below x1 indicate an overall loss), with both increases and decreases being observed. Of these, 13 classes contained both significantly increased and decreased lipid species, including PC, alkylphosphatidylcholine, alkenylphosphatidylcholine, lysophosphatidylcholine, phosphatidylethanolamine (PE), alkylphosphatidylethanolamine, PI, CE, DG, and to a lesser extent alkylphosphatidylethanolamine, sphingomyelin, and TG. Only two classes showed consistent decreases, namely ceramide and acylcarnitines, and only one class (PS) showed consistent increases. Further details can be found in Supplementary Table S3.

The pattern in the KO treatment arm was superficially very similar to that of the FO arm, both in terms of number of species with relAUCs significantly different from x1, and in terms of consistency within classes. The main differences we observed were some increases instead of decreases amongst acylcarnitines, more PC species increased, fewer alkylphosphatidylcholine species decreased, more DG species decreased, no significant changes for PS species, and sulfatides increased.

### 3.5. Treatment-Dependent Changes in Lipid Species

**Figure 5:**
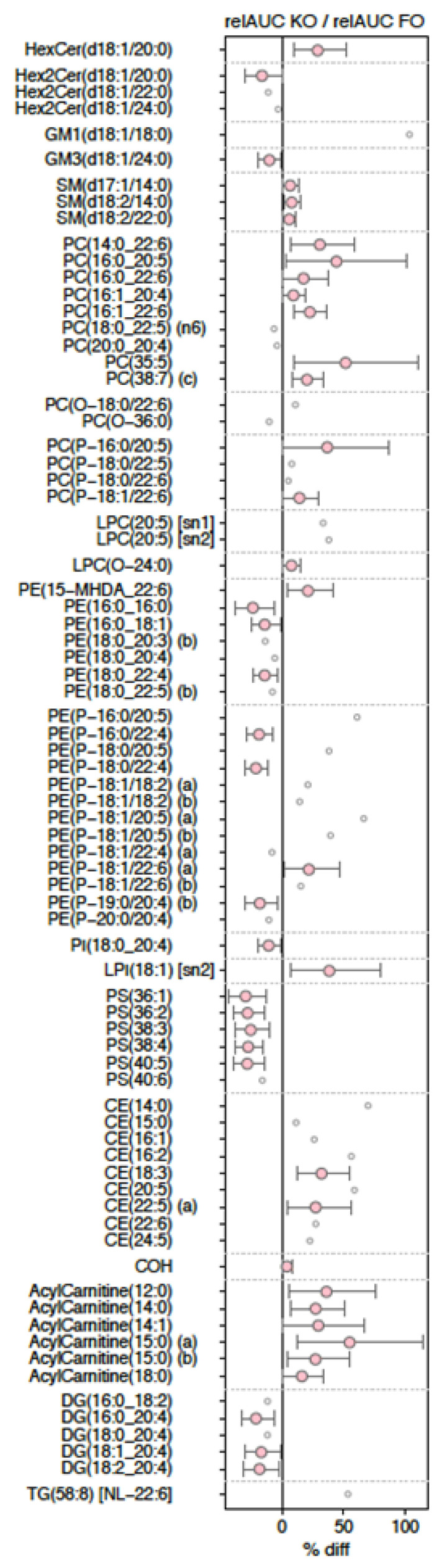
Percentage difference in relAUCs between KO and FO treatments for 77 molecular lipid species of interest.The percentage differences of the mean relAUC in KO relative to FO 30-day supplementations for 77 selected lipid molecular species are shown, with species organized by class across the y-axis. Of the selected lipids, 26 presented a nominally significantly greater relAUC in KO relative to FO, while 17 presented with a nominally significantly lower relAUC in KO relative to FO. Other selected lipids were included based on nominally significant (unadjusted *p* < 0.05) oil supplementation effects as detected by the linear mixed models. Abbreviations: FO, fish oil; KO, krill oil; HexCer, mono-hexosylceramide; Hex2Cer, di-hexosylceramide; Hex3Cer, tri-hexosylceramide; GM1, GM1 ganglioside; GM3, GM3 ganglioside; SM, Sphingomyelin; PC, Phosphatidylcholine; PC(O), alkylphosphatidylcholine; PC(P), alkenylphosphatidylcholine; LPC, lysophosphatidylcholine; LPC(O), lysoalkylphosphatidylcholine; PE, phosphatidylethanolamine; PE(P), alkenylphosphatidylethanolamine; PI, phosphatidylinositol; LPI, lysophosphatidylinositol; PS, phosphatidylserine; CE, cholesteryl ester; COH, cholesterol; DG, diacylglycerol; TG [NL], triacylglycerol (neutral loss feature).

As shown in Figure 5, there were 77 molecular lipid species that showed significant differences in their responses to the 30-day KO and FO supplementations using at least one of our analyses, i.e., lipids having a significant *p*-value for one of the treatment coefficients in the linear mixed modelling, or a nominal significance for the relAUC paired *t*-test (Supplementary Table S3).

Of these, 43 lipid species showed significant percentage differences between KO relAUC and FO relAUC (Figure 5). Twenty six of these 43 showed significantly higher relAUCs in KO compared with FO, including 10 molecular species in 3 neutral lipids classes (CE, COH, acylcarnitines) and 16 molecular species in polar lipid classes (hexosylceramide, sphingomyelin, PC, alkenylphosphatidylcholine, phosphatidylethanolamine, alkylphosphatidylethanolamine, lysophosphatidylinositol). Also, of these 26 lipid molecular species, ten (38.5%) contained omega-3 PUFA.

In contrast, 17 of 43 molecular lipid species showed significantly lower relAUC in KO compared with FO, including species in dihexosylceramide, GM3 ganglioside, phosphatidylethanolamine, alkylphosphatidylethanolamine, PI, PS, and DG (Figure 5). Of the 17 lipid species showing such differences, two species contained omega-3 PUFA, while nine (11.8%) contained omega-6 PUFA.

## 4. Discussion

This research compared the plasma lipidomic responses of 30-day supplementations of KO with FO in healthy young women. Previous studies have investigated the plasma omega-3 fatty acid levels in subjects fed KO compared with FO, but none have studied the lipidomic response over this time period. In a lipidomic analysis of the short-term postprandial responses to supplementation, we reported significant differences in the responses between KO and the FO supplementations [1]. In particular, the KO had a more pronounced effect on omega-3-containing phospholipid species, in contrast to the FO which had a more significant effect on omega-3-containing neutral lipid species.

In this longer-term study, we anticipated that there would be similar differences to those seen in the postprandial study. However, this was not the case, and for both oils the major effects were seen in numerous phospholipid species, GM3 ganglioside, hexosylceramide, acylcarnitines, CE, and DG.

Three main points can be discerned from this trial. Firstly, we found that plasma EPA levels and relAUC for EPA, but not for DHA, were significantly greater by 26.5% for KO treatment compared with the FO treatment. This could be due to greater input of EPA, reduced excretion of EPA or slower removal of EPA from the systemic circulation. Importantly, the levels of EPA in KO and FO supplements were similar (759 mg/day for KO and 786 mg/day for FO). Another input source could have been food, such that when subjects were on the KO arm of the trial, they might have consumed more foods containing EPA than when on the FO arm. However, food intake analysis via PUFA FFQ showed no significant differences in EPA-containing food intakes between the two oil supplementation periods (data not shown). It is possible that there might have been greater faecal excretion of EPA from the FO, although we did not collect faeces in this study. Typically, faecal losses of omega-3 PUFA are low [20]. The literature reports several other long-term studies comparing plasma levels of omega-3 PUFA between KO and FO treatments, and in three of these there were no significant differences in plasma EPA levels between treatments [5,6,7]. However, the study of Ramprasath et al. (four-week trial) reported significantly higher plasma EPA levels following the KO treatment compared with FO [21]. This study was criticized because the FO used was enriched in linoleic acid, confounding the interpretation of the data [22]. In all these studies, including the present study, it was difficult to exactly balance the contents of EPA and DHA between KO and FO arms, but in general, all studies had similar EPA contents. Another difference between published studies could be due to differences between supplements, in terms of the proportions of lipid classes (TG and PC in particular) in the KO [23]. In the present study, the increased plasma omega-3 levels over 30-days of KO treatment suggests a sustained maintenance of higher EPA levels due to homeostatic regulation balancing absorption into plasma with excretion in faeces and equilibration in tissues such as liver, muscle, and adipose and beta-oxidation of the EPA. It is noteworthy that the KO treatment resulted in significantly greater relAUC, compared with FO, for two phospholipid species containing EPA, namely PC 16:0–20:5 and PC(P) 16:0–20:5. The main effects of KO were on phospholipid species, especially PC, which is the main phospholipid in plasma, which remain in circulation longer than TG, which are taken up by tissues and stored or metabolized. Indeed, it has been well reported that EPA plays significant roles in various physiological functions associated with regulating blood lipids, platelet reactivity, blood pressure, and inflammation [24,25,26].

**Figure 6:**
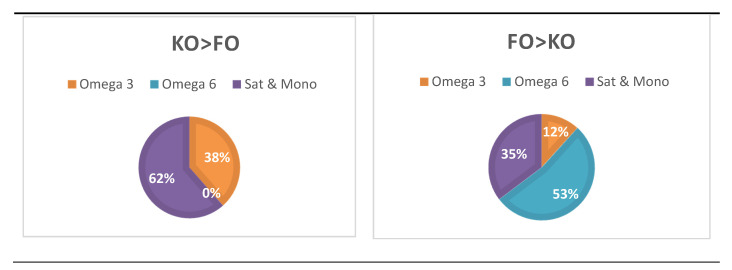
Relative proportions of omega-3 and omega-6 PUFA, and saturated and monounsaturated fatty acids based on relAUC where KO treatment was significantly greater than FO and vice versa. Data derived from Figure 5.

The second major finding was that the main significant effects of KO treatment were on omega-3-containing (such as PC(16:0–20:5)) and saturated and monounsaturated-containing molecular species (such as lysophosphatidylinositol (18:1)) compared with FO, while the main effects of FO were on omega 6-containing species (such as DG(16:0–20:4)) and saturated and monounsaturated-containing species (such as PS(36:1)), as shown in Figure 6. For example, ten of the 26 molecular species (38%), in which the KO treatment effect was significantly greater than the FO treatment, contained omega-3 PUFA and none contained omega-6 PUFA (from Figure 5). In contrast, where there was a significantly greater FO treatment effect compared with KO, only two of the 17 molecular species (12%) contained omega-3 PUFA and nine contained omega-6 PUFA (from Figure 5).

The third major finding was that use of lipidomics enabled the first clear observation of differentiation between the plasma lipid molecular species influenced by KO and FO consumption at steady state (that is, after 30 days of supplementation). This strongly suggests that the different lipid classes present in the supplements (>40% PC species for KO and >99% TG and DG for FO [1]) have a sustained influence on the lipid molecular species in plasma. To illustrate this point, the data from Figure 5 showed that KO had a significantly greater increase in 27 different lipid molecular species in seven phospholipid classes. In contrast, FO treatment showed significant effects on 17 separate lipid molecular species from four phospholipid classes (two of which were different to those influenced by KO). The physiological ramifications of these differences are not known, but they highlight that future studies on KO and FO comparisons should look beyond the omega 3 fatty acids.

We have reported that there were differences in plasma postprandial responses to KO and FO in a randomized controlled study in healthy women [1]. In the KO supplementation period, EPA and DHA were preferentially partitioned towards polar lipid molecular species including lysophospholipids, diacylphospholipids, and etherphospholipids. In contrast, in the FO supplementation period, EPA and DHA were partitioned towards TG and DG. The differences between KO and FO in the postprandial study (over five hours), in terms of the way in which EPA and DHA were incorporated into the lipidome, are likely to be contributing to the differential modification of the lipidome over the longer period.

Knowledge of metabolic fate of fatty acids from PL after digestion and absorption is considerably less than for fatty acids from TG species. It should not be concluded that fatty acids from both lipid classes simply end up being transported in chylomicrons as TG. Studies in humans using 13C-DHA-PC and 13C-DHA-TG have shown that the DHA partitioned in divergent ways into plasma, platelets, and red blood cells in terms of the time courses, lipids labelled, and turnover [27,28]. Most of the label from the 13C-DHA-TG was found in very-low-density lipoprotein-TG with lesser amount in free fatty acids (FFA); in contrast, the label from the 13C-DHA-PC was predominantly in plasma PC, lysoPC, and FFA in these short time frames (72 h). While plasma TG was labelled by both types of 13C-DHA-lipid classes, the peak of appearance of labelled TG in plasma was delayed from two hours with the 13C-TG to six hours with 13C-PC. These data illustrate the divergent metabolic processing of PUFA-related lipids derived from TG and PC. On the one hand, the expectation is that PUFA from TG will be taken up by the liver and either directed towards beta-oxidation or incorporation into lipoproteins synthesized in the liver and then exported for distribution and uptake into tissues. The contrast with PUFA from dietary PC is that there may be either direct absorption of dietary PC (as PC or lysoPC) [1] and exchange of PC with plasma lipoproteins, and also uptake of FFA and lysoPC by platelets and red blood cells, presumably by deacylation/reacylation pathway [28]. Furthermore, there is emerging evidence that albumin-bound lysoPC and even FFA might be preferred lipid carriers for uptake of DHA by the brain [29]. However, there is still insufficient evidence to know whether different lipid classes containing omega-3 PUFA, such as the case with FO and KO, have differential effects on long term health outcomes.

In terms of lipid class differences, KO supplementation resulted in a modestly higher cholesterol level and lower PS level. These differences are unlikely to have clinical significance. Rundblad et al. [30] did not find a difference in total plasma cholesterol between KO and fish-fed groups in an eight-week study, but there was a significant increase in cholesterol in the smallest very-low-density lipoprotein subclass. Future studies with KO should pay attention to possible effects on cholesterol metabolism. PS is a very minor lipid class in plasma [18] and its clinical significance in plasma is not known; however, PS is an important membrane lipid in red blood cells and neurons [31].

There have been no available reports comparing the influence of KO with FO in relation to gender and menopausal differences. Most studies comparing FO and KO have used mixed genders, often with a wide age range, and with no attempt to differentiate responses between genders. For example, in two recent studies, 18 subjects (1:1 male to female ratio) aged 18–65 years were studied by Modinger et al. [10], and 36 subjects (men and women) aged 18–70 years were studied by Rundblad et al. [30]. Therefore, it is not known whether the present results will be applicable to men and post-menopausal women.

The strength of this study is that it is the first to KO and FO supplementation in the plasma lipidome over 30 days; furthermore, it was a cross-over study. The study had several limitations, which included the small number of participants (*n* = 11), who were not being totally blinded to test oils as capsule contents had different colors (KO dark and FO light oil color). In this study, the TG analysis was not optimized for LC omega-3 PUFA. Further studies with a larger number of subjects and a more extensive lipidome measurement protocol are recommended.

## 5. Conclusions

This study shows that KO and FO do not have equivalent effects on the plasma lipidome. Plasma EPA was significantly greater following KO treatment in contrast to the FO treatment. Significant remodelling of the plasma lipidome was observed between KO and FO treatments, with a clear differentiation in their effects on different plasma lipid molecular species, with the changes in lipid species not restricted to those enriched in omega-3 PUFA. Further studies will be needed to determine whether the differences seen here have biological consequences/health benefits.

## Supplementary Materials

The following are available online at https://www.mdpi.com/2072-6643/12/9/2804/s1, Table S1: The LC omega-3 PUFA composition of study oils, Table S2: Concentration changes of lipid classes following 30-day supplementations of krill oil and fish oil, Table S3: Concentration changes of lipid species following 30-day supplementations of krill oil and fish oil.
