# Supplementary material for: Krill Oil Has Different Effects on the Plasma Lipidome Compared with Fish Oil Following 30 Days of Supplementation in Healthy Women: A Randomized Controlled and Crossover Study
Source: Nutrients. 2020 Sep 13;12(9):2804. doi: 10.3390/nu12092804 (PMC7551473; doi:10.3390/nu12092804)
Supplement: Supplementary file 1 [file nutrients-12-02804-s001.zip › HS Supplementary Table 1.docx]

Supplementary Table 1. The LC omega-3 PUFA composition of study oils

|  | **Krill oil** | | **Fish oil** | | |
| --- | --- | --- | --- | --- | --- |
|  | per capsule (mg) | **7 capsules (mg)**  **per day** | per capsule (mg) | **5 capsules (mg)**  **per day** |  |
| EPA (20:5 omega-3) | 108.4 (3.4) | **759** | 157.3 (6.4) | **786** |  |
| DPA (22:5 omega-3) | 13.4 (2.5) | **94** | 36.3 (12.2) | **182** |  |
| DHA (22:6 omega- 3) | 59.5 (2.1) | **417** | 94.6 (6.7) | **473** |  |
| Total LC omega-3 PUFA | 181.3 (2.7) | **1270** | 288.2 (8.4) | **1441** |  |

Values are expressed as mean (standard error mean) of five randomly chosen oil capsules mixed together and analysed six times using gas chromatography. The single capsule fill weight was 1.054 g for KO and 1.063 g for FO, which was used to calculate the EPA, DPA and DHA contents. The total LC omega-3 PUFA represents EPA, DHA and DPA. Abbreviations: EPA, eicosapentaenoic acid; DHA, docosahexaenoic acid; DPA, docosapentaenoic acid; LC omega-3 PUFA, long-chain omega-3 polyunsaturated fatty acids; mg, milligram.
